# Porous nitrogen-enriched carbonaceous material from marine waste: chitosan-derived carbon nitride catalyst for aerial oxidation of 5-hydroxymethylfurfural (HMF) to 2,5-furandicarboxylic acid

**DOI:** 10.1038/s41598-017-14016-5

**Published:** 2017-10-19

**Authors:** Sanny Verma, Mallikarjuna N. Nadagouda, Rajender S. Varma

**Affiliations:** 1 Oak Ridge Institute for Science and Education, P. O. Box 117, Oak Ridge, TN 37831 USA; 2 Water Systems Division, Water Resources Recovery Branch, National Risk Management Research Laboratory, U. S. Environmental Protection Agency, 26 West Martin Luther King Drive, MS 443, Cincinnati, Ohio 45268 USA

## Abstract

Chitosan-derived, porous nitrogen-enriched carbonaceous carbon nitride catalyst (PCN_x_) has been synthesized from marine waste and its use demonstrated in a metal-free heterogeneous selective oxidation of 5-hydroxymethyl-furfural (HMF) to 2,5-furandicarboxylic acid (FDCA) using aerial oxygen under mild reaction conditions.

## Introduction

**Figure 1:**
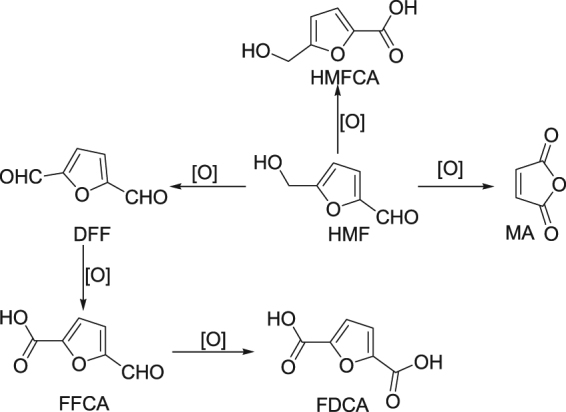
Oxidation products generated from 5-hydroxymethylfurfuryl (HMF).

Growing demand for petroleum-derived products due to the waning reserves of fossil resources has prompted researchers to seriously consider the sustainable utilization of high-value chemicals and transportation fuels from readily available biomass resources^1–6^. 5-hydroxymethylfurfuryl (HMF) is one of the common but important platform chemical derived from carbohydrates and can be further upgraded to a variety of useful entities (Fig. 1) such as 2,5-furandicarboxylic acid (FDCA), 5-hydroxymethyl-2-furancarboxylic acid (HMFCA), 5-formyl-2-furancarboxylic acid (FFCA), maleic anhydride (MA) and 2,5-diformylfuran (DFF)^7–11^. Among these, FDCA is widely used entity of significant value deployed in the production of bio-based polymers namely polyethylene 2,5-furandicarboxylate (PEF) and fine chemicals^12–14^. Interestingly, FDCA can be considered a viable substitute for the petroleum-derived terephthalic acid, which is used as an essential molecule in the synthesis of polybutyleneterephthalate (PBT) and polyethylene terephthalate (PET) plastics^15,16^.

Earlier, HMF has been fully oxidized to FDCA using toxic oxidants such as nitric acid and potassium permanganate often in stoichiometric quantities generating copious amount of wastes^17^. Various metal-based homogeneous and heterogeneous catalytic systems have been reported in literature for the direct oxidation of HMF to FDCA^18–22^. Among homogeneous catalysts, metal-bromide have been reported for the oxidation of HMF^23–25^. Yet, these catalysts are difficult to separate and frequently lead to the formation of inorganic wastes as by-products that result in environmental contamination. Heterogeneous catalysts such as Pd-, Pt-, and Au-based or bimetallic catalysts including earth abundant metal-based catalysts have also been explored for the synthesis of FDCA under aerobic conditions^26–31^. However, industrial applications of these metal-based heterogeneous catalysts has not been fruitful in view of higher catalyst cost and the leaching of metal ions into reaction systems making purification more difficult thus culminating in severe environmental pollution. Hence, the search and development of sustainable, cost-effective, metal-free, and efficient heterogeneous catalysts is actively pursued for the aerobic oxidation of HMF to FDCA.

Recently, Wu *et al*. observed the efficient oxidation of HMF to FDCA with yields of 80% using metal-free *N*-doped nanoporous graphitic (NNC) catalyst at 80 °C under aerobic condition^32^; catalyst was synthesized *via* the pyrolysis of zeolitic imidazole frameworks such as ZIF-8 at 900 °C. However, this method requires catalyst preparation *via* circuitous route and extended reaction time. In continuous of our work towards the development of sustainable protocol in synthetic transformations^33–39^, herein, we report a metal-free, efficient method for the aerial oxidation of HMF to FDCA using marine waste originated chitosan-derived porous carbon nitride (PCN_x_) catalyst as a solid catalyst.

## Synthesis and Characterization of Catalyst

**Figure 2:**
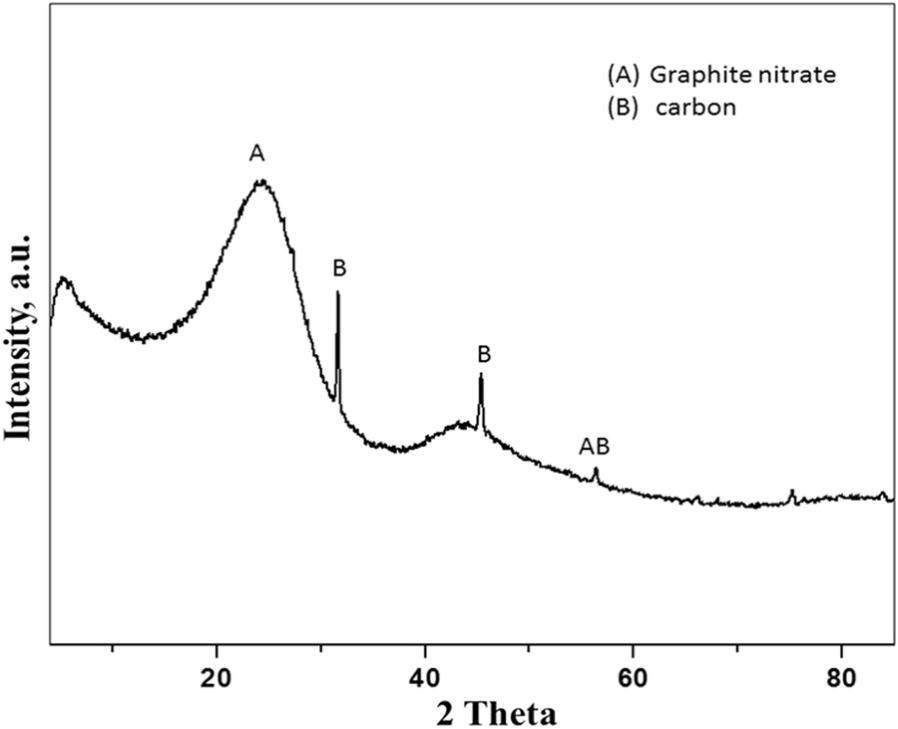
XRD analysis of PCN_x_ catalyst.

**Figure 3:**
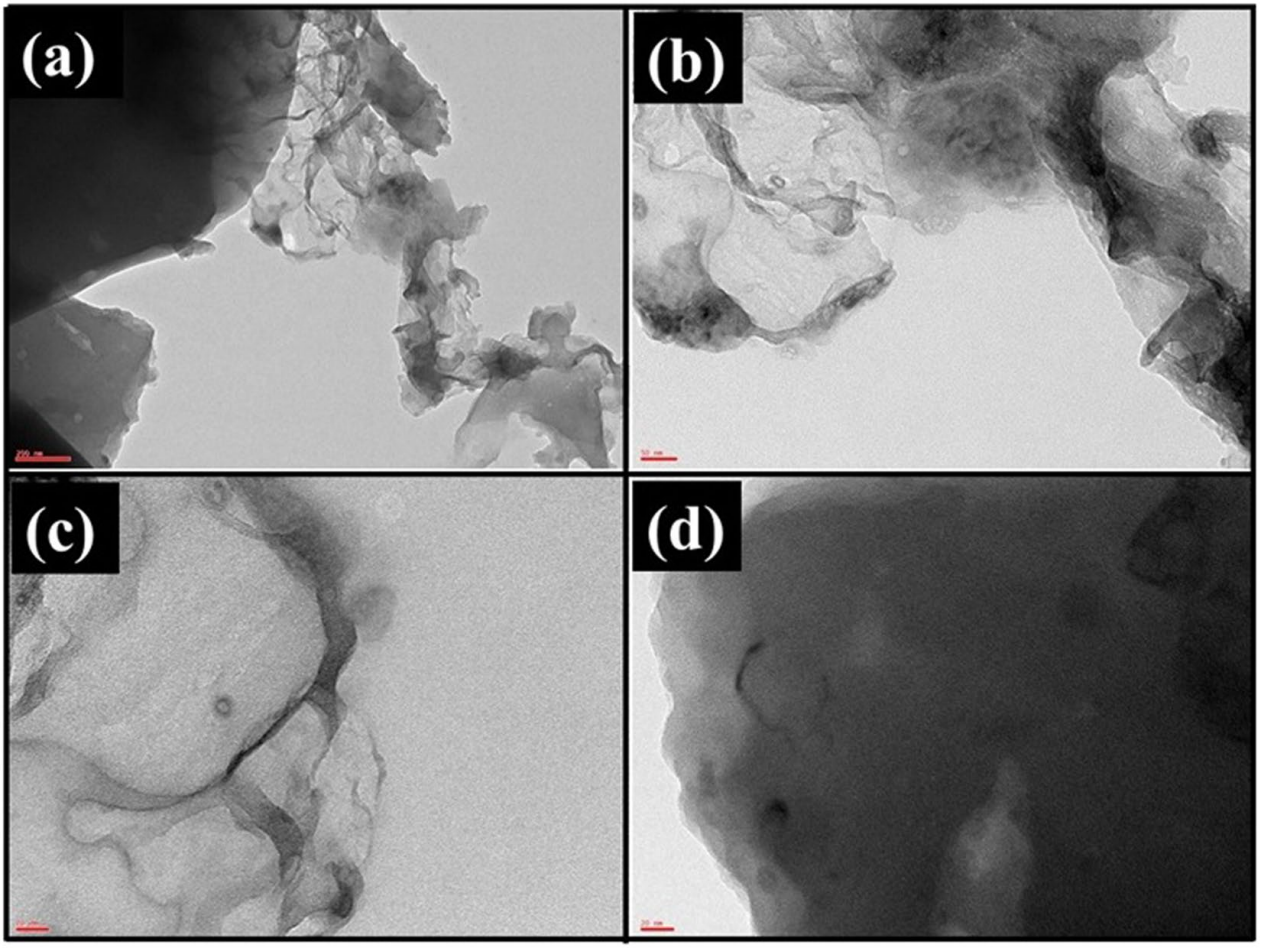
TEM analysis of PCN_x_ catalyst.

The porous carbon nitride catalyst (PCN_x_) was synthesized via calcination of chitosan at 300 °C for 4 hours under nitrogen atmosphere. The ensuing PCN_x_ catalyst was characterized using X-ray diffraction (XRD), transmission electron microscope (TEM), and Brunauer–Emmett–Teller (BET) analysis. The XRD patterns of the PCN_x_ catalyst show the characteristic pattern of graphitized carbon (Fig. 2). The graphitic line (002) of the PCN_x_ catalyst was observed at the diffraction peak of 24.97° corresponding to inter-layer spacing of about 0.345 nm which is usually attributed to a high degree of crystallinity of graphitic layers. This XRD pattern also reveals a low content of amorphous carbon and impurities. Additionally, the XRD diffraction peak of 24.97° confirms the presence of glassy carbon known as graphite nitrate supports as described by Afolabi *et al*.^40^. TEM analysis shows porous structure of PCN_x_; wrinkles and bends are easily visible in Fig. 3 which are instigated by various defects. The porous nature of the material was further supported by BET surface area analysis and was found to be 92.83 m^2^/g.

## Results and Discussion

**Figure 4:**
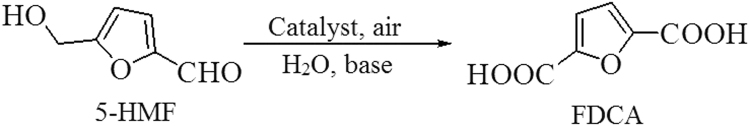
Oxidation of 5-HMF to FDCA.

To study the feasibility for the catalytic aerial oxidation of 5-HMF to FDCA conversion (Fig. 4), different metal-free carbonaceous materials under basic conditions and varying temperature range were evaluated (Table 1, entries 1–22). Various carbon-based catalysts were examined namely graphite, graphene oxide (GO), carbon nanotubes (CNT), and carbon nanofibers (CNF) for the aerial oxidation of 5-HMF to FDCA using water as a solvent and K_2_CO_3_ as a base at different temperature (Table 1, entries 1–12); no FDCA production was discerned after 36 hours of reaction (Table 1, entries 1–12). It has been reported that the graphitic nitrogen activates oxygen and plays a central role in the aerobic oxidation of alcohols^41^. Consequently, we tested *N*-doped carbon materials that contained graphitic nitrogen as shown in Table 1 (Table 1, entries 13–22). Nitrogen doped graphene gave 5% of FDCA after 36 hours (Table 1, entry 15) whereas graphitic carbon nitride (g-C_3_N_4_) afforded only 8% and 15% yields of FDCA at 50 °C and 70 °C, respectively (Table 1, entries 17–18). Nearly quantitative yield of FDCA, however, was obtained when PCN_x_ was used as a catalyst (Table 1, entries 19–22); FDCA yields of 8%, 46%, 83% were observed at 30 °C, 50 °C and 70 °C, respectively (Table 1, entries 19–21). Notably, increasing the reaction temperature to 80 °C, did not give any further improvement in yield (Table 1, entry 22). Furthermore, to understand the effect of base on the reaction, different bases such as NaOH, KOH, Na_2_CO_3_ were also evaluated at 70 °C (Table 1, entries 23–25). However, these bases failed to increase the product yield under aerial condition.

**Table 1:** Screening of catalysts and reaction optimization for FDCA conversion^a^.

Entry	Catalyst	Time	Temperature	Yield^b^
1	Graphite	36 h	30 °C	—
2	Graphite	36 h	50 °C	—
3	Graphite	36 h	70 °C	—
4	GO	36 h	30 °C	—
5	GO	36 h	50 °C	—
6	GO	36 h	70 °C	—
7	CNT	36 h	30 °C	—
8	CNT	36 h	50 °C	—
9	CNT	36 h	70 °C	—
10	CNF	36 h	30 °C	—
11	CNF	36 h	50 °C	—
12	CNF	36 h	70 °C	—
13	*N*-doped graphene	36 h	30 °C	—
14	*N*-doped graphene	36 h	50 °C	traces
15	*N*-doped graphene	36 h	70 °C	5%
16	g-C_3_N_4_	36 h	30 °C	—
17	g-C_3_N_4_	36 h	50 °C	8%
18	g-C_3_N_4_	36 h	70 °C	15%
19	PCN_x_	36 h	30 °C	8%
20	PCN_x_	36 h	50 °C	46%
21	PCN_x_	36 h	70 °C	83%
22	PCN_x_	48 h	80 °C	83%
23^c^	PCN_x_	36 h	70 °C	79%
24^d^	PCN_x_	36 h	70 °C	80%
25^e^	PCN_x_	36 h	70 °C	77%

a) Reaction condition: 5-HMF (1.0 mmol), water (10.0 ml), PCN_x_ (20 mg), K_2_CO_3_ (1.0 mmol); b) Isolated yield; c) NaOH (1.0 mmol); d) KOH (1.0 mmol); e) Na_2_CO_3_ (1.0 mmol).

### Recycling of chitosan-derived porous CN_X_ catalyst for the aerial oxidation of 5-HMF to FDCA

**Figure 5:**
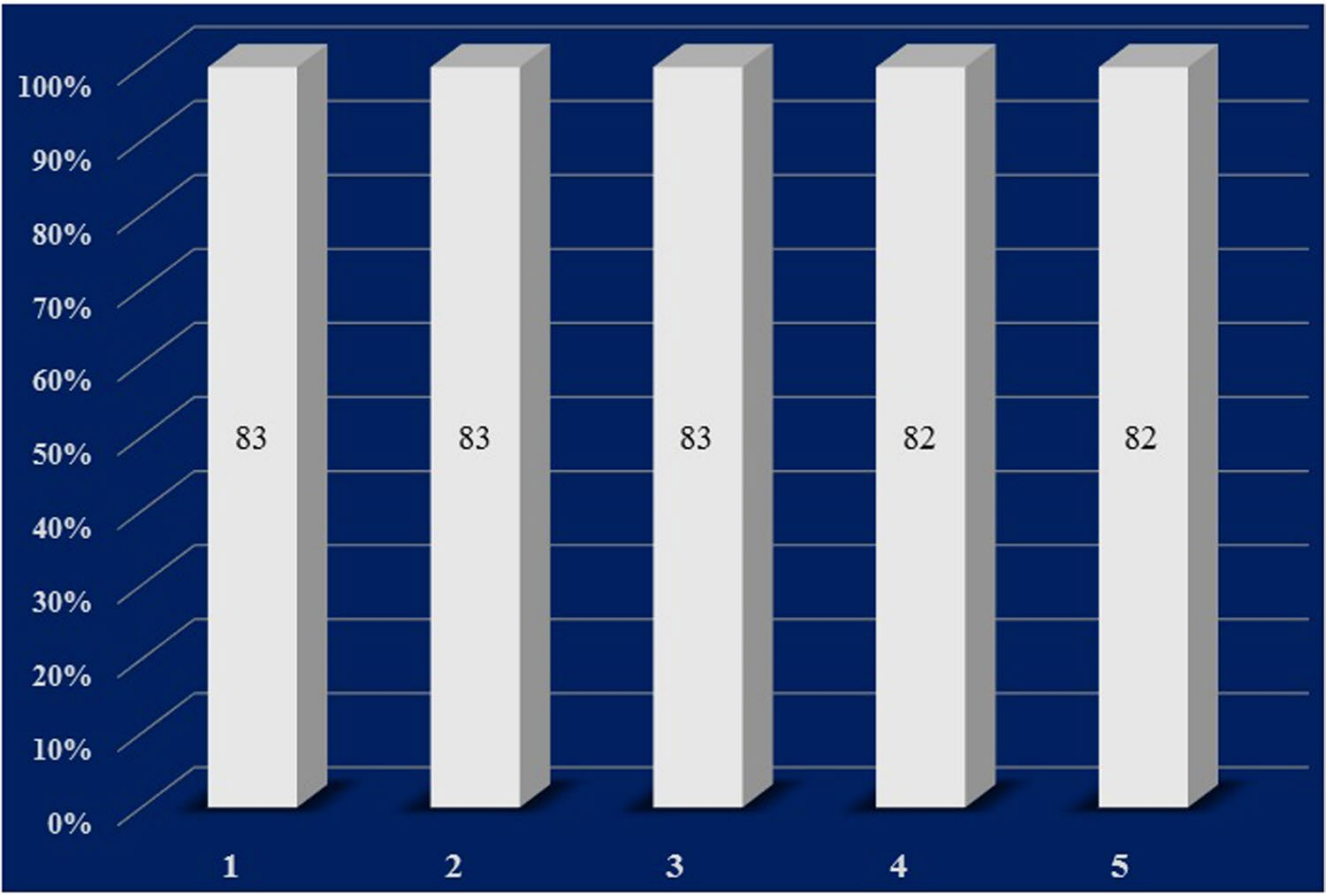
Recycling of chitosan derived porous CN_X_ catalyst.

A set of experiments were performed using 5-HMF in water. After the completion of each reaction, the CN_X_ catalyst was recovered using membrane (0.47 μm pore size) filter, washed with water and reused for the oxidation of a fresh batch of 5-HMF. The CN_X_ catalyst could be recycled and reused up to five times without any loss in its activity (Fig. 5). The XRD analysis of the CN_X_ catalyst before and after the reaction confirms that there is no significant change in the pattern/morphology of the catalyst, which signifies high stability of CN_X_ during the course of the reaction (Supporting Information).

## Conclusion

We have developed an efficient, sustainable, cost-effective and metal-free protocol for the aerial oxidation of 5-HMF to FDCA using marine waste originated chitosan-derived porous CNx catalyst under mild reaction conditions. This highly active PCNx has been synthesized *via* calcination of the chitosan at 300 °C under nitrogen atmosphere. The graphitic nitrogen in PCNx activates the oxygen and plays a key role in the aerobic oxidation of alcohols; the oxidation of 5-HMF to FDCA is accomplished in high yield (83%) under ambient air pressure at 70 °C. The PCNx catalyst shows very good recyclability and no significant loss of activity has been observed up to the fifth run.

### Disclaimer

The views expressed in this article are those of the authors and do not necessarily represent the views or policies of the U.S. Environmental Protection Agency. Any mention of trade names or commercial products does not constitute endorsement or recommendation for use.

## Electronic supplementary material

Supplementary Information
