# Supplementary material for: Porous nitrogen-enriched carbonaceous material from marine waste: chitosan-derived carbon nitride catalyst for aerial oxidation of 5-hydroxymethylfurfural (HMF) to 2,5-furandicarboxylic acid
Source: Sci Rep. 2017 Oct 19;7:13596. doi: 10.1038/s41598-017-14016-5 (PMC5648871; doi:10.1038/s41598-017-14016-5)
Supplement: Supplementary file 1 — Supplementary Information [file 41598_2017_14016_MOESM1_ESM.pdf]

Supporting Information

Porous nitrogen-enriched carbonaceous material from marine waste: chitosan-derived carbon nitride catalyst for aerial oxidation of 5-hydroxymethylfurfural (HMF) to 2,5-furandicarboxylic acid

Sanny Verma[a], Mallikarjuna N. Nadagouda[b] and Rajender S. Varma[b*]

[a]Oak Ridge Institute for Science and Education, P. O. Box 117, Oak Ridge TN, 37831, USA.

[b]Water Systems Division, Water Resources Recovery Branch, National Risk Management Research Laboratory, U. S. Environmental Protection Agency, 26 West Martin Luther King Drive, MS 443, Cincinnati, Ohio 45268, USA.

E-mail: varma.rajender@epa.gov

Contents

Procedure for the synthesis of chitosan derived PCN_X catalyst

Procedure for the aerial oxidation of 5-HMF to FDCA

XRD analysis of Recycled chitosan derived PCN_X catalyst

^1H and ^{13}C NMR spectra of FDCA

Procedure for the synthesis of chitosan-derived PCN_X catalyst

Porous CN_X catalyst was prepared *via* carbonization of chitosan at 300 °C for 4 h at a heating rate of 5°C/min under a nitrogen atmosphere. After cooling down to room temperature, CN_X catalyst was obtained as a fine brown powder. The prepared porous CN_X catalyst was further characterized by transmission electron microscopy (TEM), Brunauer–Emmett–Teller (BET) analysis, X-ray diffraction (XRD) analysis.

Procedure for the aerial oxidation of HMF to FDCA

A 25 mL three-neck round bottomed flask equipped with a magnetic stirring bar and a balloon filled with air was charged with 5-hydroxymethylfurfural (1.0 mmol), PCN_X catalyst (25 mg), K_2CO_3 (1.0 mmol) and water (10 mL). The reaction mixture was heated at 70 ^oC for 36 hours. After 36 hours, the product was cooled to room temperature and the solution was filtered using vacuum filtration assembly fitted with membrane (0.47 μm pore size). The ensuing product was analyzed using NMR.

XRD analysis of recycled chitosan-derived PCN_X catalyst

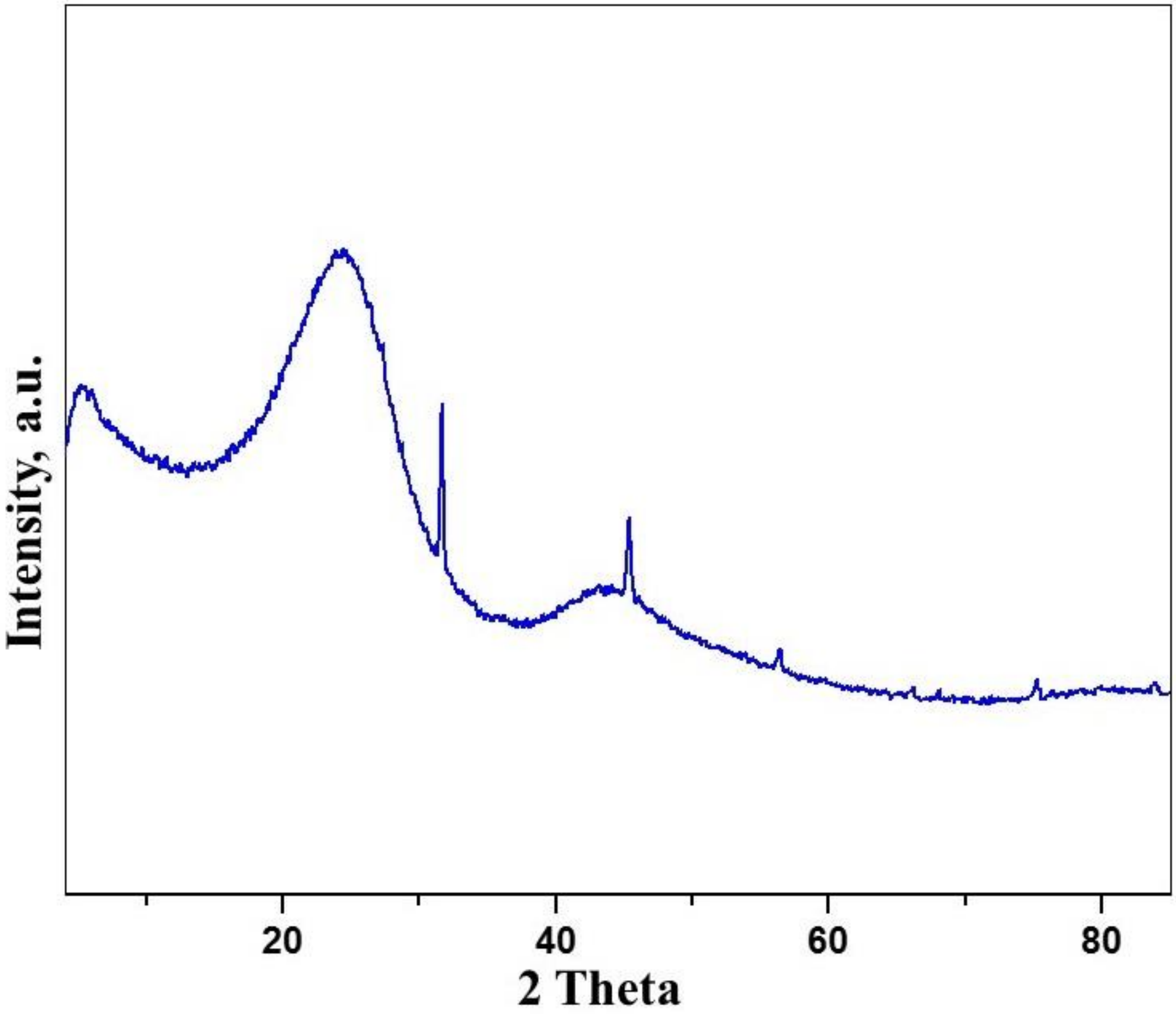

S1. XRD analysis of recycled chitosan derived PCN_X catalyst

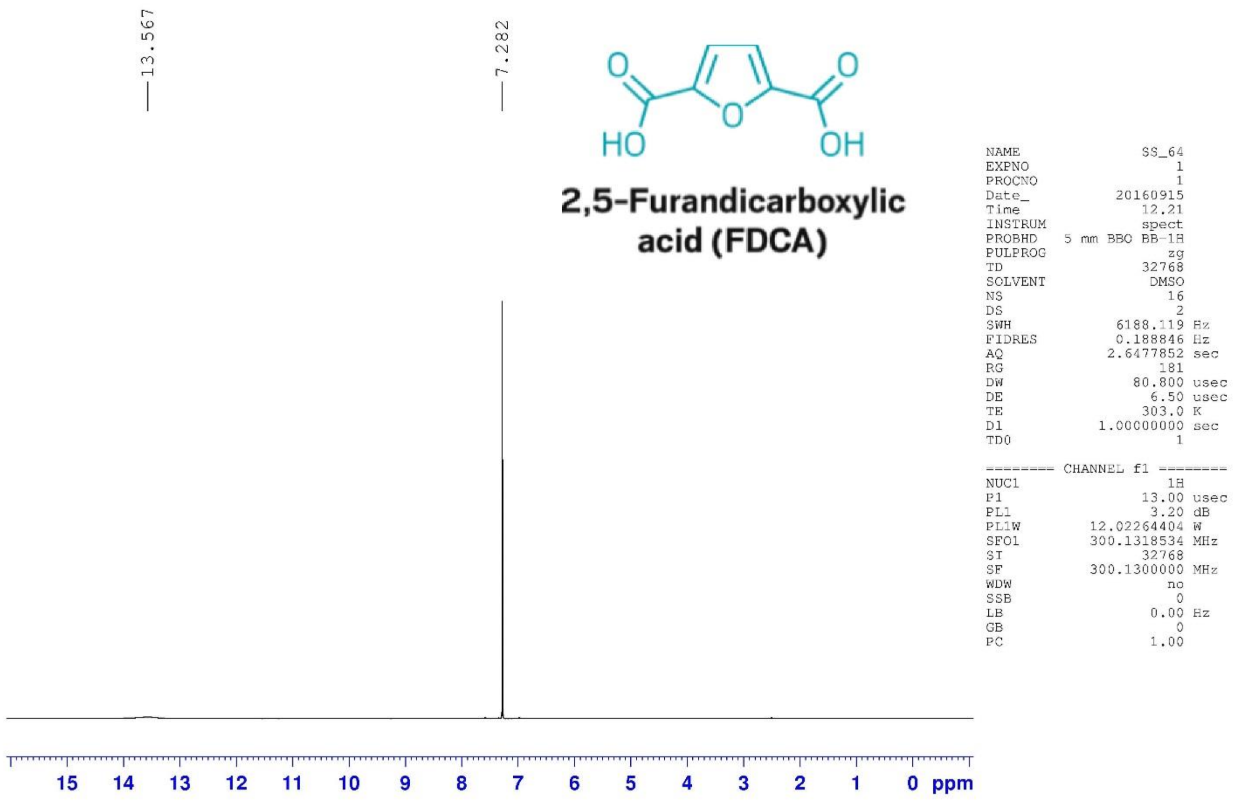

13.567
7.282
HO
OH
O
2,5-Furandicarboxylic acid (FDCA)
NAME SS_64
EXPNO 1
PROCNO 1
Date_ 20160915
Time 12.21
INSTRUM spect
PROBHD 5 mm BBO BB-1H
PULPROG zg
TD 32768
SOLVENT DMSO
NS 16
DS 2
SWH 6188.119 Hz
FIDRES 0.188846 Hz
AQ 2.6477852 sec
RG 181
DW 80.800 usec
DE 6.50 usec
TE 303.0 K
D1 1.00000000 sec
TD0 1
======== CHANNEL f1 ========
NUC1 1H
P1 13.00 usec
PL1 3.20 dB
PL1W 12.02264404 W
SFO1 300.1318534 MHz
SI 32768
SF 300.1300000 MHz
WDW no
SSB 0
LB 0.00 Hz
GB 0
PC 1.00
15 14 13 12 11 10 9 8 7 6 5 4 3 2 1 0 ppm

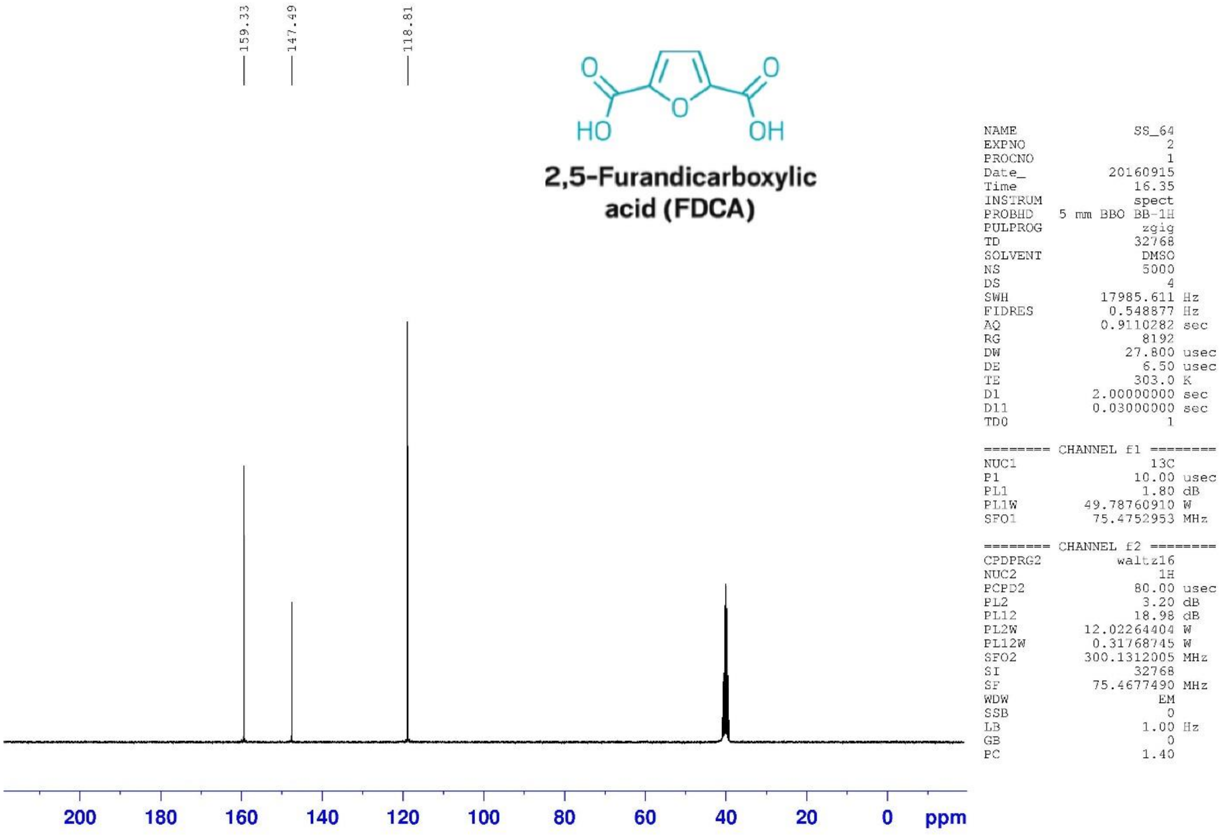
159.33
147.49
118.81
2,5-Furandicarboxylic acid (FDCA)
HO
O
OH
NAME SS_64
EXPNO 2
PROCNO 1
Date_ 20160915
Time 16.35
INSTRUM spect
PROBHD 5 mm BBO BB-1H
PULPROG zgig
TD 32768
SOLVENT DMSO
NS 5000
DS 4
SWH 17985.611 Hz
FIDRES 0.548877 Hz
AQ 0.9110282 sec
RG 8192
DW 27.800 usec
DE 6.50 usec
TE 303.0 K
D1 2.00000000 sec
D11 0.03000000 sec
TD0 1
======== CHANNEL f1 ========
NUC1 13C
P1 10.00 usec
PL1 1.80 dB
PL1W 49.78760910 W
SFO1 75.4752953 MHz
======== CHANNEL f2 ========
CPDPRG2 waltz16
NUC2 1H
PCPD2 80.00 usec
PL2 3.20 dB
PL12 18.98 dB
PL2W 12.02264404 W
PL12W 0.31768745 W
SFO2 300.1312005 MHz
SI 32768
SF 75.4677490 MHz
WDW EM
SSB 0
LB 1.00 Hz
GB 0
PC 1.40
200 180 160 140 120 100 80 60 40 20 0 ppm
